# Occurrence and prognostic effect of cervical spine injuries and cervical artery injuries with concomitant severe head injury

**DOI:** 10.1007/s00701-020-04279-9

**Published:** 2020-03-10

**Authors:** Juho Vehviläinen, Tuomas Brinck, Matias Lindfors, Jussi Numminen, Jari Siironen, Rahul Raj

**Affiliations:** 1 Department of Neurosurgery, University of Helsinki and Helsinki University Hospital, Topeliuksenkatu 5, PO. Box 266, 00029 Helsinki, Finland; 2 Department of Orthopaedics and Traumatology, University of Helsinki and Helsinki University Hospital, Helsinki, Finland; 3 Department of Radiology, University of Helsinki and Helsinki University Hospital, Helsinki, Finland

**Keywords:** Head injury, Traumatic brain injury, Blunt cerebrovascular injury, Cervical spine injury, Cervical trauma

## Abstract

**Background:**

Blunt cerebrovascular injuries (BCVIs) and cervical spinal injuries (CSIs) are not uncommon injuries in patients with severe head injury and may affect patient recovery. We aimed to assess the independent relationship between BCVI, CSI, and outcome in patients with severe head injury.

**Methods:**

We identified patients with severe head injury from the Helsinki Trauma Registry treated during 2015–2017 in a large level 1 trauma hospital. We assessed the association between BCVI and SCI using multivariable logistic regression, adjusting for injury severity. Our primary outcome was functional outcome at 6 months, and our secondary outcome was 6-month mortality.

**Results:**

Of 255 patients with a cervical spine CT, 26 patients (10%) had a CSI, and of 194 patients with cervical CT angiography, 16 patients (8%) had a BCVI. Four of the 16 BCVI patients had a BCVI-related brain infarction, and four of the CSI patients had some form of spinal cord injury. After adjusting for injury severity in multivariable logistic regression analysis, BCVI associated with poor functional outcome (odds ratio [OR] = 6.0, 95% CI [confidence intervals] = 1.4–26.5) and mortality (OR = 7.9, 95% CI 2.0–31.4). We did not find any association between CSI and outcome.

**Conclusions:**

We found that BCVI with concomitant head injury was an independent predictor of poor outcome in patients with severe head injury, but we found no association between CSI and outcome after severe head injury. Whether the association between BCVI and poor outcome is an indirect marker of a more severe injury or a result of treatment needs further investigations.

**Electronic supplementary material:**

The online version of this article (10.1007/s00701-020-04279-9) contains supplementary material, which is available to authorized users.

## Introduction

Blunt cerebrovascular injuries (BCVIs) are a non-penetrating injury to the carotid and/or vertebral artery that may cause stroke in trauma patients [5]. The occurrence of BCVI in trauma patients has varied in literature between 0.08 and 1.2% [1, 30, 36, 40]. Higher (9.2%) occurrence among traumatic brain injury (TBI) patients has been reported [12]. There have been also findings that a lack of adequate imaging techniques could give too low incidence and incidences of 2.7–6.7% among trauma patients have been reported [4, 13].

The occurrence of cervical spine injuries (CSIs) ranges between 4 and 12.5% in TBI patients [4, 11, 13, 16, 17, 19, 24, 38, 40, 41]. The overall occurrence of CSI in trauma patients is around 4% [19]. Carotid artery injuries (CAI) and vertebral artery injuries (VAI) seem to be different entities in terms of risk factors and patient outcomes. CSI correlates to VAI in cervical hyperextension injuries at high impact energies [34]. Up to 70–78% of VAI patients have a simultaneous CSI [3, 8, 34]. Correlating injuries and predisposing conditions to CAI are facial injuries, diffuse axonal injury (DAI), Glasgow Coma Scale (GCS) < 6, and road traffic collisions [34, 40]. In terms of patient outcome, CAI seem to be worse than VAI [10].

Stroke rate among BCVI patients has a large variation from 1% up to 53.8%. The majority of these strokes were already in progress when the patient has been admitted to a hospital [5, 10, 12, 17, 26, 30, 39, 40, 42].

Treatment of BCVI using either antiplatelet or anticoagulation medication seems to be equal [10]. However, there seems to be indications that the stroke rate in BCVI patients is around 10% in average regardless of any kind of intervention. This supports the theory that most of the strokes due to BCVI have already realized before admission to hospital [17, 26].

There have been efforts to construct evidence-based guidelines for treating BCVI patients [5, 9, 31]. According to guidelines, the indication criteria for computed tomography angiography (CTA) of cervical arteries would use extended Denver protocol, which includes signs and symptoms for BCVI, e.g., focal neurological deficit, expanding cervical hematoma, neurological deficit inconsistent of head computed tomography (CT), and stroke on CT/magnetic resonance imaging (MRI), or risk factors of BCVI, e.g., high-energy trauma, facial fractures, CSI, and TBI with thoracic injuries [9, 13, 14]. If these conditions are satisfied, the patient should go through CT angiography, and if BCVI is found, an antithrombotic treatment should be started as early as possible for minimum of 3 months. In addition, after 1 week and 3 months, a CT angiography control should be done [5]. Still, it seems that more rigorous screening of BCVI might not help. Even though more BCVI cases were found, it did not improve patient prognosis [18]. Also, a high number, up to 45%, of false positive BCVI in CTA have been reported in initial imaging [35].

The aims of this study were to investigate the association between cervical BCVI, CSI, and outcome in patients with severe head injury. Furthermore, we aimed to establish the occurrence of cervical BCVI and CSI in patients with severe head injury treated in the largest Finnish trauma center. We hypothesized that the presence of a cervical BCVI or CSI would not affect outcome after severe head injury and that the occurrence of cervical BCVI and CSI in this Finnish cohort of severe head injury patients would be lesser than in previously reported studies from settings outside the Nordics. The rationale for this hypothesis is that severe head injuries in Finland are often the result of low-energy trauma mechanisms among elderly patients [27, 33].

## Methods and materials

### Study setting and study population

We conducted a retrospective study of the prospectively data collecting Helsinki Trauma Registry (HTR), which has been described in detail previously [21]. The local research committee approved of the study (HUS/175/2016). We obtained through the HTR data on severely injured patients, New Injury Severity Score (NISS) > 15, treated from May 2015 through May 2017. HTR is a local trauma registry in the trauma unit of the Helsinki University Hospital, which centralizes the treatment of severe blunt injuries among adult patients (≥ 16 years) with a catchment area of 1.8 million inhabitants in southern Finland.

The trauma unit of Helsinki University Hospital implemented a routine contrast CT imaging of cervical vessels of trauma alert patients in May 2015. This allowed us to investigate the occurrence of blunt vascular trauma in the cervical area in addition to the lesions in cervical spine.

We included patients with a severe head injury (head Abbreviated Injury Score (AIS) ≥ 3) [15]. We screened all patients who underwent a whole-body contrast CT including the cervical arteries and all patients who underwent a non-contrast CT scan of the cervical spine.

### Definition of covariates

We assessed the severity of the CSI according to the AIS classification [15]. We further divided those with CSI patients into two groups: surgically treated and non-surgically treated CSI.

We used the Biffl grades for classification of cervical BCVI [2]. All cervical artery CT angiograms were primarily assessed by specialized radiologists. We checked the reports of all scans and re-reviewed (authors JN and RR) those with a confirmed or suspected cervical BCVI. If a lesion in cervical arteries was found, later head CT or/and MRI scans of head were screened for cerebral ischemia. We defined a brain infarction if there were signs of a new ischemic lesion in the territory of the affected cervical artery.

We defined the GCS score as the first reliable post-resuscitation GCS score prior to intubation or sedation. We defined hypotension and hypoxia as a prehospital, admission, or emergency room systolic blood pressure < 90 mmHg and an oxygen saturation < 90%. The head CT images were analyzed using Marshall CT classification [28]; in addition, epidural hematomas (EDH) and traumatic subarachnoid hemorrhages (tSAH) were identified in the images.

### Definition of outcomes

Our aim was to assess the association between cervical BCVI, CSI, and outcome after severe head injury. Our primary outcome was 6-month functional outcome, and our secondary outcome was 6-month mortality. We defined functional outcome according to the Glasgow Outcome Score (GOS) [25]. We assessed GOS from various electronic health care records (including neurosurgical and neurological follow-up visits, rehabilitation visits, neuropsychological visits, primary health care visits) retrospectively at 6 months’ time of the initial injury. We defined a GOS of 1–3 (death to severe disability) as unfavorable outcome and a GOS of 4–5 (moderate disability to good recovery) as favorable outcome. Data regarding 6-month mortality was obtained through the population registry and available for all patients.

### Statistical analyses

We used IBM SPSS Statistics version 24 for Windows (IBM Corp.) for the statistical analyses. Categorical variables are compared using a two-sided *χ*^2^ test. Continuous data were tested for skewness using the Kolmogorov-Smirnov test. Non-parametric data were compared using a Mann-Whitney *U* test, and normally distributed data were compared using an independent *t* test.

To assess the independent association between cervical BCVI and CSI, we adjusted for brain injury severity, using an IMPACT extended-based model. We included all predictors from the IMPACT extended model and calibrated the predictors’ coefficients to our dataset using multivariable logistic regression (LR) with 6-month outcome as the outcome. We assessed the area under the receiver operating curve (AUC) to assess the performance of the IMPACT extended model to adjust for case-mix. We then used the IMPACT extended model to adjust for injury severity in separate multivariable LR model with cervical BCVI and CSI as separate predictors.

Regarding missing data, we used a multiple imputation with fully conditional specification Markov Chain Monte Carlo (MCMC) method with 10 iterations. The IMPACT extended predictors that had missing values were hypoxia, hypotension, and pupil light reaction. The predictors for the MCMC analysis were age, Marshall CT classification, epidural hematoma, traumatic SAH, systolic blood pressure, oxygen saturation, NISS, and death within 30 days. Little’s MCAR test yielded a *p* value of 0.65, indicating randomly missing data.

## Results

### Baseline characteristics

**Fig. 1:**
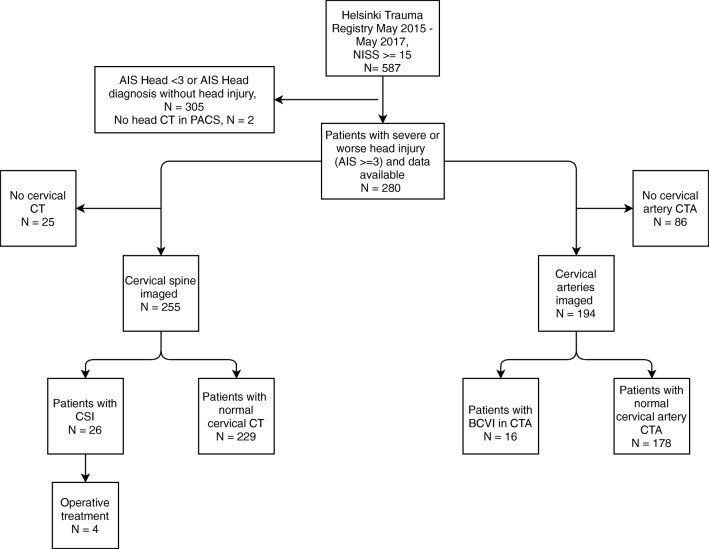
Flow chart of the patients in the study. AIS Head ≥ 3 is defined as severe head trauma. AIS, Abbreviated Injury Score; BCVI, blunt cerebrovascular injury; CSI, cervical spine injury; CT, computed tomography; CTA, computed tomography angiography; NISS, New Injury Severity Score; PACS, picture archiving and communication systems

A total of 280 patients with severe head injury were identified from the HTR during the study period (Fig. 1). Of these, 255 patients (91%) had a cervical spine CT performed and 194 patients (69%) had a cervical CT angiography performed (all patients with a cervical CTA had also a cervical spine CT performed).

Table 1 shows the patient demographics in patients who underwent CT of the cervical spine. Differences between those with and without CSI were found in injury type (traffic accidents have the highest CSI occurrence),and Marshall CT classification.

**Table 1:** Patient demographics for all 255 patients who underwent a CT of the cervical spine according to the presence of cervical spine injury status

Variable	All CT spine (*N* = 255)	CSI (*N* = 26)	No CSI (*N* = 229)	*p* value
Age, median (IQR)	52 (35–66)	45 (35–55)	53 (35–67)	0.094
Gender (% males)	76	85	75	0.282
ASA				0.169
1	97 (38%)	13 (50%)	84 (37%)	
2	104 (41%)	12 (46%)	92 (40%)	
3	46 (18%)	1 (4%)	45 (20%)	
4	5 (2%)	0	5 (2%)	
Missing	3 (1%)	0	3 (1%)	
Injury type				0.022*
High fall	45 (18%)	4 (15%)	41 (18%)	
Low fall	69 (27%)	1 (4%)	68 (30%)	
Traffic, motor vehicle	47 (18%)	9 (35%)	38(17%)	
Traffic, bicycle	21 (8%)	5 (19%)	16 (7%)	
Traffic, pedestrian	13 (5%)	3 (12%)	10 (4%)	
Other	27 (11%)	1 (4%)	26 (11%)	
Unknown	33 (13%)	3 (12%)	30 (13%)	
GCS				0.730
3–8	131 (51%)	13 (50%)	118 (52%)	
9–13	55 (22%)	3 (12%)	52 (23%)	
14–15	62 (24%)	8 (31%)	54 (24%)	
Missing	7 (3%)	2 (8%)	5 (2%)	
Motor score				0.297
1–2	89 (35%)	7 (27%)	82 (36%)	
3–4	37 (15%)	6 (23%)	31 (14%)	
5–6	118 (46%)	11 (42%)	107 (47%)	
Missing	11 (4%)	2 (8%)	9 (4%)	
Pupils				0.356
Responsive	138 (54%)	18 (69%)	120 (52%)	
Unilateral unresponsive	17 (7%)	1 (4%)	16 (7%)	
Bilateral unresponsive	69 (27%)	5 (19%)	64 (28%)	
Missing	31 (12%)	2 (8%)	29 (13%)	
Cardio-pulmonary system				
Hypotension	17 (7%)	3 (12%)	14 (6%)	0.280
Missing	6 (2%)	1 (4%)	5 (2%)	
Hypoxia	7 (3%)	1 (4%)	7 (3%)	0.369
Missing	7 (3%)	0	6 (3%)	
Marshall CT classification				0.003*
I	16 (6%)	3 (12%)	13 (6%)	
II	124 (49%)	19 (73%)	105 (46%)	
III	15 (6%)	3 (12%)	12 (5%)	
IV	15 (6%)	0	15 (7%)	
V + VI	85 (33%)	1 (4%)	84 (37%)	
tSAH	152 (60%)	16 (62%)	136 (59%)	0.832
EDH	32 (13%)	1 (4%)	31 (14%)	0.157
IMPACT extended risk for 6-month mortality (IQR)	39.7% (19.1–75.3%)	23.4% (16.4–60.0%)	45.9% (19.1–79.8%)	0.094
IMPACT extended risk for 6-month unfavorable outcome (IQR)	65.9% (39.1–91.0%)	45.8% (34.3–82.0%)	71.8% (39.1–93.0%)	0.094
Missing values for IMPACT calculation	37 (15%)	2 (8%)	35 (15%)	
NISS, median (IQR)	33.0 (26.0–43.0)	34.0 (24.5–39.5)	33.0 (26.0–43.0)	0.610
Outcome 6 months				0.401
Dead	62 (24%)	5 (19%)	57 (25%)	
GOS 2–3	37 (15%)	4 (15%)	33 (14%)	
GOS 4–5	123 (48%)	12 (46%)	111 (48%)	
Missing	33 (13%)	5 (19%)	28 (12%)	

*Abbreviations*: *CSI* cervical spine injury, *CT* computed tomography, *CTA* computed tomography angiography, *GCS* Glasgow Coma Score, *tSAH* traumatic subarachnoidal hemorrhage, *EDH* epidural hematoma, *NISS* New Injury Severity Score, *GOS* Glasgow Outcome Score, *Hypotension* systolic BP < 90, *Hypoxia* SpO2 < 90%. If GCS or pupil response was missing at the time of hospital admission, prehospital values were used

*Statistically significant *p* < 0.05

Table 2 shows the patient demographics in patients who underwent a CTA of the cervical arteries. There were no statistically significant differences in baseline characteristics between those with and without BCVI.

**Table 2:** Patient demographics for all 194 patients who underwent a CT angiography of the cervical arteries according to the presence of blunt cervical vascular injury (BCVI)

	All CT cervical arteries (*N* = 194)	BCVI (*N* = 16)	No BCVI (*N* = 178)	*p* value
Age, median (IQR)	48 (32–64)	50 (32–62)	48 (32–65)	0.773
Gender (% males)	76	88	75	0.253
ASA				0.701
1	86 (44%)	9 (56%)	77 (43%)	
2	77 (40%)	6 (38%)	71 (40%)	
3	25 (13%)	1 (6%)	24 (14%)	
4	3 (2%)	0	3 (2%)	
Missing	3 (2%)	0	3 (2%)	
Injury type				0.388
High fall	41 (21%)	3 (19%)	38 (21%)	
Low fall	29 (15%)	1 (6%)	28 (16%)	
Traffic, motor vehicle	47 (24%)	6 (38%)	41 (23%)	
Traffic, bicycle	21 (11%)	3 (19%)	18 (10%)	
Traffic, pedestrian	12 (6%)	2 (13%)	10 (6%)	
Other	20 (10%)	0	19 (11%)	
Unknown	24 (12%)	1 (6%)	23 (13%)	
GCS				0.559
3–8	98 (51%)	10 (63%)	88 (49%)	
9–13	42 (22%)	2 (13%)	40 (22%)	
14–15	49 (25%)	3 (19%)	46 (26%)	
Missing	5 (3%)	1 (6%)	4 (2%)	
Motor score				0.328
1–2	70 (36%)	9 (56%)	61 (34%)	
3–4	25 (13%)	1 (6%)	24 (13%)	
5–6	90 (46%)	5 (31%)	85 (48%)	
Missing	9 (5%)	1 (6%)	8 (5%)	
Pupils				0.562
Responsive	112 (58%)	11 (69%)	101 (57%)	
Unilateral unresponsive	15 (8%)	1 (6%)	14 (8%)	
Bilateral unresponsive	43 (22%)	2 (13%)	41 (23%)	
Missing	24 (12%)	2 (13%)	22 (12%)	
Cardio-pulmonary system				
Hypotension	15 (8%)	3 (19%)	12 (7%)	0.067
Missing	2 (1%)	1 (6%)	1 (1%)	
Hypoxia	37 (19%)	4 (25%)	33 (19%)	0.637
Missing	21 (11%)	3 (19%)	18 (10%)	
Marshall CT classification				0.432
I	16 (8%)	2 (13%)	14 (8%)	
II	113 (58%)	9 (56%)	104 (58%)	
III	13 (7%)	2 (13%)	11 (6%)	
IV	13 (7%)	2 (13%)	11 (6%)	
V + VI	39 (20%)	1 (6%)	38 (21%)	
tSAH	115 (59%)	12 (75%)	103 (58%)	0.181
EDH	23 (12%)	1 (6%)	22 (12%)	0.469
IMPACT extended risk for 6-month mortality (IQR)	33.7% (13.9–70.3%)	55.3% (10.8–80.7%)	33.7% (15.5–70.3%)	0.368
IMPACT extended risk for 6-month unfavorable outcome (IQR)	59.5% (29.9–88.5%)	78.6% (23.7–93.4%)	59.5% (32.7–88.5%)	0.368
Missing values for IMPACT calculation	25 (13%)	2 (13%)	23 (13%)	
NISS, median (IQR)	33.5 (27.0–43.0)	38.0 (27.0–43.0)	33.0 (26.0–43.0)	0.136
Outcome 6 months				0.053
Dead	41 (21%)	8 (50%)	33 (19%)	
GOS 2–3	26 (13%)	3 (19%)	23 (13%)	
GOS 4–5	101 (52%)	4 (25%)	97 (54%)	
Missing	26 (13%)	1 (6%)	25 (14%)	

*Abbreviations*: *CSI* cervical spine injury, *CT* computed tomography, *CTA* computed tomography angiography, *GCS* Glasgow Coma Score, *tSAH* traumatic subarachnoidal hemorrhage, *EDH* epidural hematoma, *NISS* New Injury Severity Score, *GOS* Glasgow Outcome Score, *Hypotension* systolic BP < 90, *Hypoxia* SpO2 < 90%. If GCS or pupil response was missing at the time of hospital admission, prehospital values were used

*Statistically significant *p* < 0.05

Online Resource 1 describes the patient demographics for the whole dataset (*N* = 280) and for sub-groups in which the cervical spine (*N* = 25) or the cervical arteries (*N* = 86) were not imaged. The main difference between the whole dataset and the not-imaged sub-groups was that patients in not-imaged sub-groups had more frequently low energy traumas.

Online Resource 2 describes the mean values of the non-imputed and imputed datasets. The imputation did not notably affect the mean values of the imputed variables.

### Cervical spine injury

Of the 255 patients who underwent CT of the cervical spine, 26 patients (10%) were diagnosed with a CSI. Four out of 26 CSI patients (15%) underwent operative treatment. Four CSI patients had varying types of spinal cord injuries ranging from small epidural hematoma to complete cord injury. One patient with complete cord injury and BCVI (Biffl grade 4) died. One patient had a favorable neurological outcome, two patients were from different hospital districts, and no follow-up neurological outcome information was available. These two patients were, however, alive within 6 months of the injury.

### Blunt cervical vascular injury

Of the 194 patients who underwent CTA of the cervical arteries, BCVI was found in 16 patients (8%, Online Resource 3). Almost two thirds of the BCVI were in the carotid arteries. There were a total of four BCVI-related brain infarctions (all carotid artery). Three out of 4 patients with a BCVI-related brain infarction died within 6 months, and the remaining patient had an unfavorable functional outcome (Online Resource 3).

### Multivariable analysis

The AUC for the IMPACT extended model for predicting 6-month mortality was 0.90–0.91 (95% CI 0.86–0.96, depending on the imputed dataset), and the AUC for predicting 6-month functional status was 0.86 (95% CI 0.81–0.91, depending on the imputed dataset), thus providing excellent case-mix adjustment.

After adjusting for case-mix, the presence of a CSI did not associate with 6-month unfavorable outcome (OR = 2.09, 95% CI 0.67–6.54, *p* = 0.205) or 6-month mortality (OR = 1.89, 95% CI 0.49–7.33, *p* = 0.358).

After adjusting for case-mix, the presence of a BCVI significantly associated with 6-month unfavorable outcome (OR = 5.99, 95% CI 1.35–26.51, *p* = 0.018) and 6-month mortality (OR = 7.90, 95% CI 1.99–31.43, *p* = 0.003).

## Discussion

In this retrospective study investigating the association between CSI, BCVI, and outcome in patients with severe head injury, we found that BCVI with concomitant severe head injury was associated with unfavorable outcome and adds to 6-month mortality. CSI on the other hand did not affect patient outcome or 6-month mortality. Of included patients, 8.2% had a diagnosed BCVI and 10.2% a diagnosed CSI. Thus, contrary to our hypothesis, the occurrences of BCVI and CSI were rather similar in our setting compared to other settings [12, 16].

Also, contrary to our hypothesis, we found that BCVI was associated with an approximately 6 times higher odds for poor functional outcome and 8 times higher odds for 6-month mortality. Still, there was no difference in the severity of brain injury (according to the IMPACT extended model) between those with and without BCVI.

There are several reasons why BCVI may lead to poorer outcome after severe head injury. BCVI-related infarcts are the obvious reason. In our cohort, four out of 16 patients had a diagnosed BCVI-related infarction, all of which had a poor outcome. Due to the skewed distribution of BCVI in the outcome groups (all patients with BCVI-related infarct had an unfavorable outcome), it was not possible to assess the independent association between BCVI-related brain infarction and outcome. Yet, it is plausible to relate these infarcts to poorer outcome. Still, one should notice that half of the patients with a BCVI-related infarct underwent MRI. It is possible that whether all patients would have undergone brain MRI scans, the occurrence of BCVI-related brain infarcts would have been higher. It is also possible that the BCVI patients received earlier and more aggressive antithrombotic medication therapies, which may increase the risk of intracranial hemorrhage progression. For 12 BCVI patients, low molecular weight heparin (LMWH) was initiated on post-traumatic days 1 to 5, and for four BCVI patients, LMWH could not or had not been initiated due to hemorrhage progression and/or dismal prognosis. Out of the four BCVI patients with a diagnosed brain infarction, LMWH was initiated on post-traumatic days 1 to 2 for two patients, and for two patients, LMWH was not initiated due to the latter aforementioned reason. Importantly, patients with BCVI were more often subject to high-energy traumas. Thus, it is likely that patients with concomitant severe head injury and BCVI really had more severe injuries than those without a BCVI, despite of similar IMPACT extended model predictions, which might fail to adjust for the full spectrum of differences in injury severity.

According to our hypothesis, we did not find any association between CSI and outcome. Again, there were no differences in injury severity as measured by the IMPACT extended model between those with and without a CSI. Like the BCVI patients, those with CSI were more frequently in a high-energy trauma than those without CSI. However, this did not reflect in poorer outcomes. Of those with CSI, only four had a documented spinal cord injury. It is possible that CSI may have been an indirect marker of poor outcome, whether the prevalence of spinal cord injury would have been higher. Other studies have found that those with CSI and spinal cord injury have poorer outcomes than those CSI without spinal cord injury, which is in line with our findings [20, 32].

Currently, there are evidence-based guidelines for the treatment of spontaneous carotid dissections [6, 29]; however, in terms of BCVI, the treatment guidelines are still in progress. The choice between antiplatelet versus anticoagulation and the time for optimal medication start remain unknown. Based on this retrospective series, it is not possible to draw any conclusions but only to highlight the relatively strong association between BCVI and outcome. Whether this is due to the BCVI itself, higher trauma energies and more severe injuries, or possible negative effects of the treatment requires further studies, and due to the relative rareness of BCVI, probably multicenter collaborative projects.

Regarding the occurrence of BCVI, likely, the most wide-spread recommendation to screen for BCVI is the extended Denver protocol [9, 13, 14]. Other screening recommendations are, for example, modified Memphis criteria [7]. For CSI, the de facto screening criteria have been the National Emergency X-Radiography Utilization Study (NEXUS) criteria and the Canadian C-spine rules [22, 23, 37]. The local protocol used in HTR is in accordance with these screening protocols, and the occurrence of BCVI and CSI in our cohort was similar to that of previous studies (9.2% for BCVI [12] and 4–12.5% for CSI [4, 11, 13, 16, 17, 19, 24, 38, 40, 41]), indicating appropriate usefulness of the screening guidelines.

### Strengths and limitations

This study is a cohort study including consecutive patients admitted to the trauma unit of Helsinki University Hospital (catchment area of almost 2 million for severe head injury), which is one of the largest trauma units in the Nordic countries. Despite our study being retrospective in nature, the high-quality electronic patient registries enable excellent follow-up in combination with extensive and validated data from the HTR [21].

Still, there are limitations that should be addressed. First, we had a relatively small number of both BCVIs and CSIs. Thus, the statistical analyses may be somewhat underpowered (e.g., association between BCVI-related brain infarction and outcome). Second, not all patients underwent cervical CT or CTA. Thus, we were forced to separate these groups from one another. Still, there were no major differences in patient characteristics between those with and without complete imaging studies. Thus, we do not believe that this affects our results. Third, we do not routinely screen BCVI patients for brain infarcts using MRI. Still, eight out of 16 patients with a BCVI underwent a brain MRI at some point after the trauma. Thus, it is possible that clinically silent infarcts may have been undiagnosed, underestimating the occurrence of BCVI-related infarcts. Third, although CTA of the cervical arteries and CT of the spine were part of the routine trauma protocol for severe head injury patients during the study period, 9% of patients did not undergo a cervical spine CT and 31% of patients did not undergo a CTA of the cervical arteries. Reasons for protocol deviations require further investigation. Fourth, the possibility of false positives for diagnosing BCVI has been described to be as high as 45% [35]. We re-reviewed the images of patients with a radiological or clinical suspicion of BCVI. Thus, it is possible that there were patients with an initially misdiagnosed or silent BCVI that we did not pick up. However, whether these misdiagnosed or silent BCVI are clinically meaningful remains unknown, but we do not believe this to affect our results.

### Summary

We found that BCVI with concomitant head injury was an independent predictor of poor outcome in patients with severe head injury, but we found no association between CSI and outcome after severe head injury. The occurrence of BCVI in our study was 8% that is similar to previous studies in patients with severe head injury [12]. Whether the association between BCVI and poor outcome is an indirect marker of a more severe injury or a result of treatment needs further investigations.

## Electronic supplementary material

ESM 1 (PDF 56 kb)

ESM 2 (PDF 59.8 kb)

ESM 3 (PDF 81 kb)
