# Supplementary material for: Occurrence and prognostic effect of cervical spine injuries and cervical artery injuries with concomitant severe head injury
Source: Acta Neurochir (Wien). 2020 Mar 10;162(6):1445–53. doi: 10.1007/s00701-020-04279-9 (PMC7235059; doi:10.1007/s00701-020-04279-9)
Supplement: Supplementary file 1 — (PDF 56 kb) [file 701_2020_4279_MOESM1_ESM.pdf]

Online Resource 1: Patient demographics for the whole dataset (N=280) and for the groups that did not go through CTA of cervical arteries (N=86) or native CT of the cervical spine (N=25).

	ALL (280)	CTA of cervical arteries not imaged (86)	CT of spine not imaged (25)
Age median (IQR)	53 (36-67)	57 (46-73)	59 (25.5-38.0)
Gender (% males)	77	79	84
**ASA**			
1	99 (35%)	13 (15%)	2 (8%)
2	116 (41%)	39 (45%)	12 (48%)
3	57 (20%)	32 (37%)	11 (44%)
4	5 (2%)	2 (2%)	0
Missing	3 (1%)	0	0
**Injury type**			
High fall	45 (16%)	4 (5%)	0
Low fall	88 (31%)	59 (69%)	19 (76%)
Traffic, motor vehicle	48 (17%)	1 (1%)	1 (4%)
Traffic, bicycle	21 (8%)	0	0
Traffic, pedestrian	13 (5%)	1 (1%)	0
Other	28 (10%)	8 (9%)	1 (4%)
Unknown	27 (13%)	13 (15%)	4 (16%)
**GCS**			
3-8	139 (50%)	41 (48%)	8 (32%)
9-13	60 (21%)	18 (21%)	5 (20%)
14-15	72 (26%)	23 (27%)	10 (40%)
Missing	9 (3%)	4 (5%)	2 (8%)
**Motor score**			
1-2	96 (34%)	24 (28%)	5 (20%)
3-4	38 (14%)	13 (15%)	1 (4%)
5-6	134 (48%)	44 (51%)	16 (64%
Missing	12 (4%)	5 (6%)	3 (12%)
**Pupils**			
Responsive	149 (53%)	37 (43%)	11 (44%)
Unilateral unresponsive	19 (7%)	4 (5%)	2 (8%)
Bilateral unresponsive	78 (28%)	33 (38%)	7 (28%)
Missing	34 (12%)	12 (14%)	5 (20%)
**Cardio-pulmonary system**			
Hypotension	19 (7%)	2 (2%)	0
Missing	4 (1%)	4 (5%)	0
Hypoxia	9 (3%)	3 (4%)	2 (8%)
Missing	6 (2%)	6 (7%)	0
**Marshall CT classification**			
I	16 (6%)	0	0
II	128 (46%)	15 (17%)	4 (16%)
III	18 (6%)	5 (6%)	3 (12%)
IV	16 (6%)	3 (4%)	1 (4%)
V+VI	102 (36%)	63 (73%)	17 (68%)
tSAH	116 (59%)	51 (59%)	14 (56%)
EDH	34 (12%)	11 (13%)	2 (8%)
Missing	0	0	0
Impact Extended	8.0 (5.3)	9.0 (5.0)	8.5 (4.6)
Missing	77 (28%)	33 (38%)	13 (52%)

NISS median (IQR)	33.0 (26.0-42.3)	33.5 (25.0-41.3)	34.0 (25.5-38.0)
**Outcome 6 months**			
Dead	76 (27%)	35 (41%)	14 (56%)
GOS 2-3	38 (14%)	12 (14%)	1 (4%)
GOS 4-5	130 (46%)	29 (34%)	7 (28%)
Missing	36 (13%)	10 (12%)	3 (12%)
Abbreviations: , CT = Computer Tomography, CTA = Computed Tomography Angiography; GCS = Glasgow Coma Score; tSAH = traumatic Subarachnoidal Haemorrhage; EDH = Epidural Haematoma; NISS = New Injury Severity Score; GOS = Glasgow Outcome Score; Hypotension = Systolic BP < 90; Hypoxia = SpO2 < 90%; If GCS or pupil response was missing at time of hospital admission, prehospital values were used.			
