# Supplementary material for: Occurrence and prognostic effect of cervical spine injuries and cervical artery injuries with concomitant severe head injury
Source: Acta Neurochir (Wien). 2020 Mar 10;162(6):1445–53. doi: 10.1007/s00701-020-04279-9 (PMC7235059; doi:10.1007/s00701-020-04279-9)
Supplement: Supplementary file 2 — (PDF 59.8 kb) [file 701_2020_4279_MOESM2_ESM.pdf]

Online Resource 2: Mean values and standard deviations of non-imputed and imputed variable used for IMPACT extended based model.

**Cervical arteries imaged (N = 194)**	Mean values (Std)	Mean values (Std)
	non-imputed	imputed
Motor score	3.65 (2.15)	3.62 (2.14)
Pupils	0.594 (0.867)	0.605 (0.868)
Hypoxia (prehospital and hospital data combined)	0.214 (0.411)	0.250 (0.433)
Hypotension (prehospital and hospital data combined)	0.102 (0.304)	0.114 (0.319)
**Cervical spine imaged (N = 255)**		
	non-imputed	imputed
Motor score	3.67 (2.14)	3.65 (2.16)
Pupils	0.692 (0.913)	0.692 (0.910)
Hypoxia (prehospital and hospital data combined)	0.202 (0.402)	0.243 (0.429)
Hypotension (prehospital and hospital data combined)	0.0885 (0.285)	0.0988 (0.299)
