# Supplementary material for: Occurrence and prognostic effect of cervical spine injuries and cervical artery injuries with concomitant severe head injury
Source: Acta Neurochir (Wien). 2020 Mar 10;162(6):1445–53. doi: 10.1007/s00701-020-04279-9 (PMC7235059; doi:10.1007/s00701-020-04279-9)
Supplement: Supplementary file 3 — (PDF 81 kb) [file 701_2020_4279_MOESM3_ESM.pdf]

Online Resource 3: Characteristics of the Blunt Cervical Vascular Traumas (BCVIs) on 16 patients.

**Biffl grade**	**N (%)**	**Brain infarction (N)**
1	4 (21%)	1 (25%)
2	10 (53%)	2 (20%)
3	0 (0%)	0 (0%)
4	4 (21%)	1 (25%)
5	1 (5%)	0 (0%)
**Location**	**N (%)**	**Brain infarction (N)**
CAI	11 (58%)	4 (36%)
VAI	8 (42%)	0 (0%)

We defined a brain infarction if there were signs of a new ischemic lesion in the territory of the affected cervical artery. For example, one patient with an internal carotid artery and a vertebral artery BCVI may get two brain infarctions.

Please note that one patient had bilateral BCVI and in addition two injuries on the right side. Two patients with BCVI Biffl grade 2 and 4 died within one day and no control images were available. Also, two patients with Biffl grade 2 and 4 lesions were missing control CT scans of the head and possible infarction could not be verified. This patient's primary head CT was normal.

Abbreviations: CAI = Carotid Artery Injury, VAI = Vertebral Artery Injury
